# Molecular Dynamic Simulation and Experiment Validation on the Diffusion Behavior of Diffusion Welded Fe-Ti by Hot Isostatic Pressing Process

**DOI:** 10.3390/ma16165626

**Published:** 2023-08-15

**Authors:** Cheng Gu, Sheng Zeng, Weili Peng, Guoqiang You, Jianhua Zhao, Yajun Wang

**Affiliations:** 1 College of Materials Science and Engineering, Chongqing University, Chongqing 400045, China; 201809131156@cqu.edu.cn (S.Z.); ygq@cqu.edu.cn (G.Y.);; 2 National Engineering Research Center for Magnesium Alloys, Chongqing University, Chongqing 400044, China

**Keywords:** diffusion behavior, Fe-Ti interface, hot isostatic pressing process, molecular dynamic simulation

## Abstract

A reliable bonding interface between steel and Ti alloy is required for producing a steel/Ti bimetal composite. In this study, molecular dynamic simulations and diffusion welding experiments using the hot isostatic pressing process were conducted to study the atomic diffusion at the Fe-Ti interface. The simulation results indicate that the diffusion layer thickness is thinner in single crystals compared to polycrystals at the same temperature. This difference may be explained by polycrystals having grain boundaries, which increase atomic disorder and facilitate diffusion. The radial distribution function (RDF) curves for Fe-Fe and Ti-Ti exhibit a similar pattern over time, with a main peak indicating the highest atom density within a specific radius range and relatively strong binding between the central atoms and their nearest neighbors. The observed changes in the diffusion coefficient with temperature in the simulations align well with the experimental results. This study enhances the understanding of Fe-Ti interface diffusion mechanism and provides valuable insights for broader applications of steel/Ti bimetal composites.

## 1. Introduction

Titanium alloys are extensively utilized for their lightweight nature, excellent mechanical properties, and good corrosion resistance [1,2,3]. However, the high production cost of Ti alloys restricts their usage primarily to high-tech fields such as aerospace, nuclear, and marine engineering [3,4]. On the other hand, structural steels offer high strength at a much lower cost. By combining Ti alloys with structural steels, bimetal composites can be produced at a reduced cost, which is advantageous for promoting their wide application [5]. To achieve this, a reliable Fe-Ti bonding interface is necessary [6]. Various welding methods were employed for this purpose, including ultrasonic welding [7], laser welding [8], friction welding [9], and diffusion welding [5].

Lin et al. [6] utilized ultrasonic welding to weld Ti and steel and analyzed changes in interface microstructure and bonding strength. Zhang et al. [8] employed laser welding to join Ti alloy and stainless steel, without filler metal, and then they used Ta/V/Fe [10] as an interlayer to produce a joint between Ti and stainless steel. A maximum tensile strength of 627 MPa was reported with a Ta/V/Fe interlayer. Meshram et al. [11] investigated friction welding of a Fe-Ti joint. Gotawala and Shrivastava [12] conducted an analysis on the evolution of intermetallic compounds during friction stir welding of stainless steel and pure titanium without an interlayer. Cheepu and Susila [9] used a Ta interlayer to investigate the friction welding between Ti and stainless steel. Rodmacq et al. [13] produced Fe/Ti multilayers by triode sputtering with an average composition of Fe33Ti67. Chen et al. [14] used a copper filler to join Ti alloys with stainless steel by MIG–TIG welding. Liu et al. [15] performed TIG welding on a Ta15 alloy with 18Cr8Ni stainless steel, with Cu alloys as filler metals. Chu et al. [16] developed a quantitative relation between the microstructure and mechanical properties of Ti/Fe bimetal bonded with Cu20V filler. Ramirez [17] produced Ti-clad steel welded joints with different interlayers of Ni-Ti, NiCu-Ti, and NiCr-Ti. Pugacheva et al. [18] studied the microstructure of a Fe/Ti welded joint with Cu/Ta interlayer. Wu and Yang [19,20] investigated a Ti/steel interface during a TIG welding thermal cycling. Cui et al. [21] produced a composite plate made of Ti alloy and low alloy steel by explosive welding. Most of the above researches were performed by adding an interlayer between Ti and steel. This approach is necessary because insufficient bonding strength in a Ti/steel composite can result from interface defects. Intermetallic compound formation at the interface is the primary cause of these defects, which can limit the interface bonding of a composite. The presence of more types of interface compounds has a greater impact on the interface bonding strength.

To comprehend the atom diffusion at the Fe-Ti interface, researchers have employed simple diffusion studies and utilized various characterization methods. Balogh et al. [22] concluded that the Fe-on-Ti and Ti-on-Fe interfaces of Ti/Fe/Ti trilayers have different interface widths and alloy compositions. Li et al. [5] used polycrystalline α-Ti samples to study the TiC layer growth at the interface between Ti and steel. They found that the activation energy for the growth of TiC was higher in V. Additionally, simulation methods are considered as powerful tools to study interface evolution. Prasanthi et al. [23] studied the diffusion of Fe atoms in a Ti lattice through molecular dynamics (MD) simulation. Xiang et al. [24] examined the Fe-Ti interface diffusion in Ti/steel composite plates by theory and simulation methods. However, most of these studies assume a single-crystal system while most samples are polycrystalline in nature, and the effects of pressure have not been taken into consideration. Furthermore, limited experimental validation was assessed when simulations were conducted.

This article presents a study on the Fe-Ti interface diffusion behavior through MD simulations and experimental diffusion welding of Fe-Ti joints. The simulation results were validated by comparing them with experimental findings. Furthermore, the correlation between calculated diffusion coefficient and diffusion temperature was discussed, providing insights into the Fe-Ti interface diffusion mechanism.

## 2. Materials and Methods

### 2.1. Simulation Method

**Figure 1:**
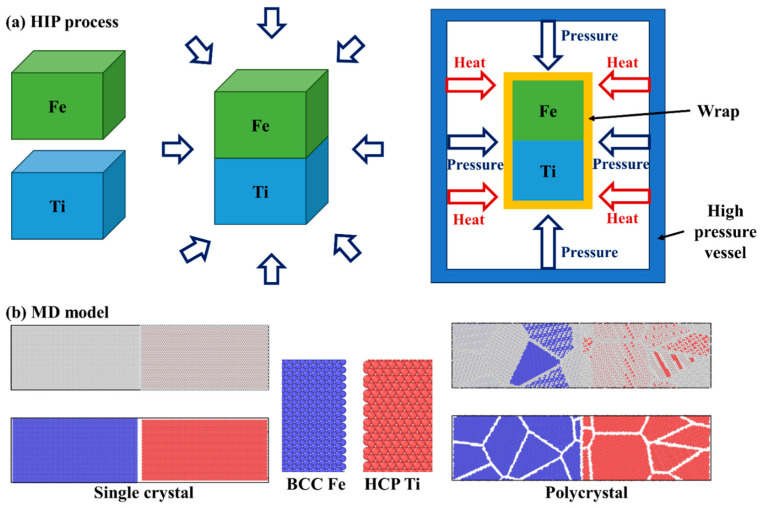
(**a**) Illustration of hot isostatic pressing process, and (**b**) molecular dynamic model of single-crystal and polycrystal Fe-Ti diffusion couple.

We employed the large-scale atomic molecular massively parallel simulator (LAMMPS) [25] to investigate atom diffusion through molecular dynamics simulations in this study. The Ti alloy and steel were considered as either single crystals or polycrystals. The single-crystal Ti exhibited hexagonal close-packed (HCP) lattices with a lattice constant of approximately 2.95 Å, while the single-crystal Fe had body-centered cubic (BCC) lattices with a lattice constant of around 2.86 Å. Atomsk version beta-0.12 software and the Voronoi method [26] were used to create the polycrystal Ti and Fe, with 5 grains and a grain size of about 4.6 nm. In Figure 1b, the molecular dynamic model is depicted, where blue atoms represent Fe atoms and red atoms represent Ti atoms. For both single-crystal and polycrystal cases, the initial simulation region had dimensions of 400 Å × 100 Å × 100 Å, containing a total of 133,640 Ti atoms and 170,100 Fe atoms. A time step of 0.001 ps was used in the simulations.

At the beginning, the Fe and Ti models were separated by a 2 Å gap. Both models underwent complete relaxation using an NPT ensemble (ensuring that the number of atoms, pressure, and temperature of the system remained unchanged) at different temperature (1123 K and 1223 K) for 500 ps to reach a stable state, with initial atom velocities following the Maxwell distribution. The two models were then merged, employing shrinking boundaries in the diffusion direction (*Z* axis) and periodic boundaries in the other directions (*X* axis and *Y* axis). The system was set to an NVT ensemble (ensuring that the number of atoms, volume, and temperature of the system remained unchanged), and the system temperature was kept constant by means of the Nosé–Hoover method. An external pressure of 30 MPa was applied to the entire system throughout the simulation.

The accuracy of the potential function used in the model, which describes the interactions between atoms or molecules, is vital in determining the reliability of the MD simulation results. Baskes et al. [27] proposed an extension of the embedded-atom method (EAM) known as modified embedded-atom method (MEAM), which can be expressed as:

(1) $$E=\underset{i}{\sum }\left\{F_{i}\left(\vec{\rho_{i}}\right)+\frac{1}{2}\underset{j\neq i}{\sum }\phi_{ij}\left(R_{ij}\right)\right\}$$

(2) $$F_{i}\left(\vec{\rho }\right)=A_{i}E_{i}^{0}\vec{\rho_{i}}ln\vec{\rho_{i}}$$

where E represents the overall system energy, $F_{i}$ denotes the embedding function, $\rho_{i}$ is the background electron density, $\phi_{ij}\left(R_{ij}\right)$ refers to the pair interaction potential, $R_{ij}$ is the separate distance, $A_{i}$ stands for the atomic structure parameter, and $E_{i}^{0}$ is the binding energy of atom i. To describe interatomic interactions in the Fe-Ti binary system, the MEAM interatomic potential developed by Sa and Lee [28] was adopted in this study.

### 2.2. Diffusion Welding Experiment

In the diffusion welding experiments conducted in this study, commercially pure titanium (CP-Ti, provided by Shuoer Metal Materials Co., Ltd., Xingtai, China) and 316 stainless steel (provided by Dongguan Sanjian Metal Materials Co., Ltd., Dongguan, China) were used, with the chemical compositions specified in Table 1. Samples of both titanium and steel for diffusion bonding were cut into dimensions of 8 mm × 8 mm × 5 mm using electrical discharge machining (EDM). The surfaces intended for connection were treated by grinding and polishing before being pressed together and fixed. To facilitate atomic diffusion at the titanium/steel interface, a hot isostatic pressing (HIP) process [29,30] was performed as depicted in Figure 1a. Following the HIP process, the samples were furnace-cooled. The HIP parameters involved two treatment temperatures of 850 and 950 °C, a pressure of up to 30 MPa, and a holding time of 40 min. After the diffusion welding experiment, the samples underwent grinding and polishing for microstructure characterization. The microstructure at the titanium/steel interface was investigated using scanning electron microscopy (SEM, JEOL JSM-7800F), while the chemical composition distributions were analyzed via energy-dispersive spectroscopy (EDS).

## 3. Results and Discussion

### 3.1. MD Simulation Results

**Figure 2:**
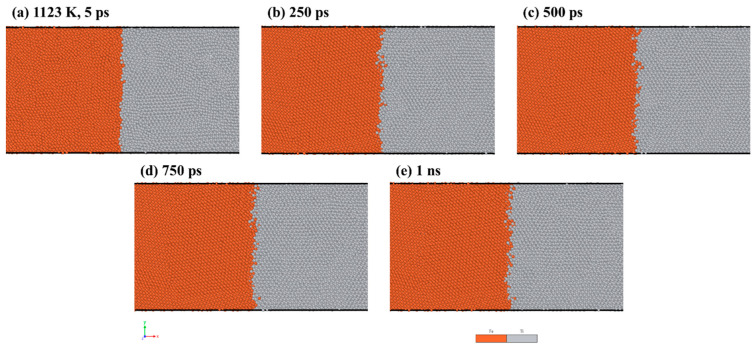
MD simulation results of Fe/Ti interface atom distribution at 1123 K for: (**a**) 5 ps, (**b**) 250 ps, (**c**) 500 ps, (**d**) 750 ps, and (**e**) 1 ns.

To visualize the atom configurations, we utilized the open-source software OVITO version 3.8.5 [31]. Figure 2 shows the results of the MD simulations, displaying the distribution of atoms at the Fe/Ti interface at a temperature of 1123 K over different holding times. As depicted in Figure 2a, there was limited diffusion of Fe and Ti atoms into each other after 5 ps. Nevertheless, with an increased diffusion time of 1 ns, more interdiffusion between Fe and Ti atoms started to occur. Additionally, the interface between Fe and Ti shrank with increasing diffusion time. This is due to a binding energy forming when the two layers contacted, thereby strengthening the diffusion between atoms.

**Figure 3:**
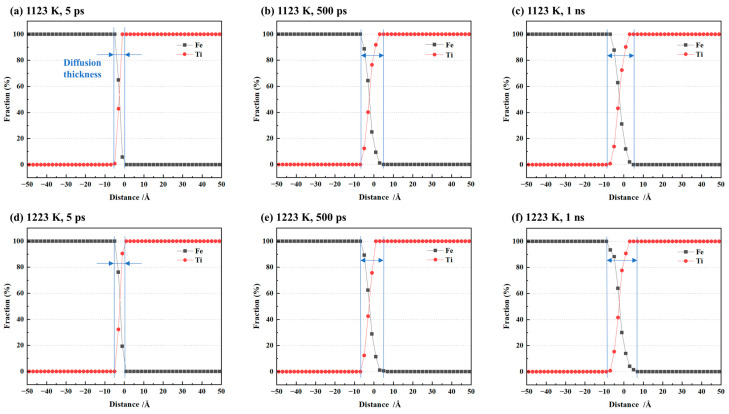
Atomic concentration distribution of single-crystal cases at (**a**) 1123 K for 5 ps, (**b**) 1123 K for 500 ps, (**c**) 1123 K for 1 ns, (**d**) 1223 K for 5 ps, (**e**) 1223 K for 500 ps, and (**f**) 1223 K for 1 ns.

To quantitatively analyze the Fe/Ti interface diffusion, we measured the diffusion layer thickness at the Fe/Ti interface by calculating the diffusion distance. We also recorded the atomic concentration distribution along the diffusion direction at different diffusion times, as illustrated in Figure 3a–c. The diffusion layer became thicker with time. Additionally, simulations were also conducted at a temperature of 1223 K, and the corresponding atomic concentration distributions are presented in Figure 3d–f. It can be observed that the diffusion layer thickness at 1223 K was relatively larger compared to that at 1123 K.

**Figure 4:**
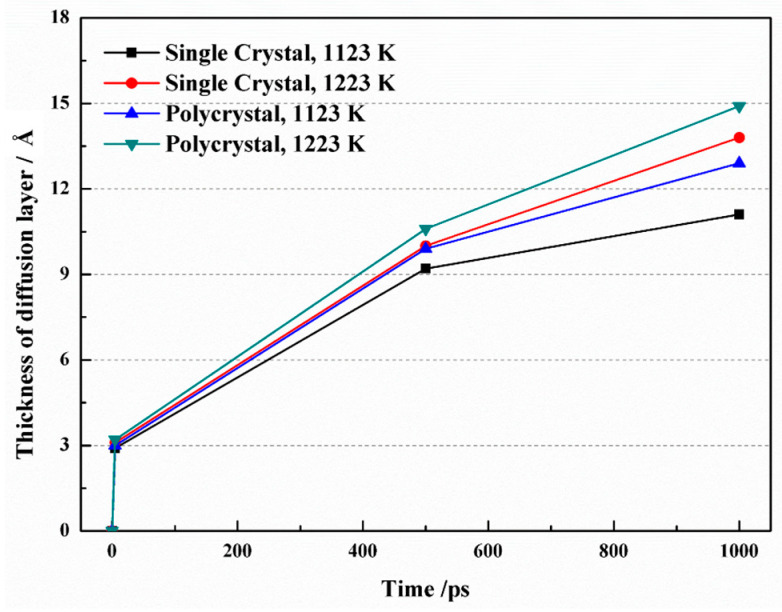
Diffusion layer thickness as a function of time at different simulation conditions.

Figure 4 illustrates the growth of the diffusion layer thickness over time under different simulation conditions. Initially, the thickness increased rapidly, but the rate of increase gradually slowed down. In the case of a single crystal, as the time raised from 5 ps to 1 ns at a temperature of 1123 K, the thickness of the diffusion layer expanded from 9.22 Å to 11.08 Å. At 1223 K, the thickness increased from 10.04 Å to 13.79 Å over the same time interval. Interestingly, when comparing the same temperature, the diffusion layer was thinner in single crystals compared to polycrystals. This discrepancy may be attributed to the presence of grain boundaries in polycrystals, which introduce disorder among atoms and facilitate their diffusion.

The radial distribution function (RDF, g(r)) is a valuable tool for assessing the level of disorder in a system by quantifying the likelihood of locating an atom at a specific distance, r, from another atom. It offers insights into interatomic relationships and interaction strengths within the system, providing valuable information for studying atomic structures and gaining a deeper understanding of their properties.

**Figure 5:**
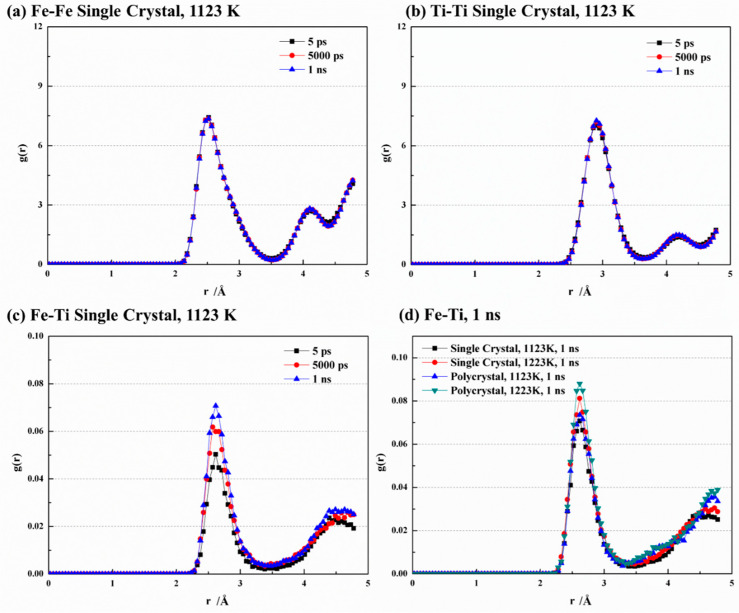
Comparison of RDF curves at different conditions of (**a**) Fe-Fe, (**b**) Ti-Ti, (**c**) Fe-Ti, and (**d**) Fe-Ti at 1 ns.

Figure 5 showcases the radial distribution function (RDF) curves for Fe-Fe, Ti-Ti, and Fe-Ti under different conditions. At 1123 K, the RDF curves of Fe-Fe and Ti-Ti exhibit remarkable similarity at various time points (Figure 5a,b). They both display a dominant peak that indicates the highest number density of atoms within a specific radius range, implying a strong binding between central atoms and their nearest neighbors. Furthermore, a secondary peak is observed at larger distances, suggesting an ordered crystal structure for both Fe and Ti. The main peak of Fe-Fe is located at 2.52 Å, indicating the nearest neighbor atoms of Fe appear at this distance and allowing for the determination of the Fe-Fe bond length. Similarly, the main peak of Ti-Ti appears at 2.91 Å, enabling the determination of the Ti-Ti bond length. These findings align with the MEAM interatomic potential [28] utilized in this study, which provides equilibrium nearest-neighbor distances of 2.48 Å and 2.92 Å for Fe and Ti, respectively. In Figure 5c, the main peak of Fe-Ti is observed at 2.61 Å, which falls within the range between Fe-Fe and Ti-Ti. Sa and Lee [28] also reported an equilibrium nearest-neighbor distance of 2.58 Å for Fe-Ti. The peak values for Fe-Fe and Ti-Ti are significantly larger compared to Fe-Ti due to stronger bonding between like atoms. The peak strength of Fe-Ti also increases with time due to the migration of crystal atoms, resulting in a disordered structure and weakened bond strength between like atoms. Conversely, during bond formation between Fe and Ti atoms, the increased bonding strength leads to higher peak values. Figure 5d compares the RDF curves of Fe-Ti with various temperatures and crystal structures. It is observed that increasing temperature leads to an increase in the peak value of Fe-Ti. In addition, the peak value is higher in the polycrystal structure than in the single-crystal structure, indicating that higher temperatures and finer grains enhance the disorder of the crystal.

### 3.2. Diffusion Experiment Results

**Figure 6:**
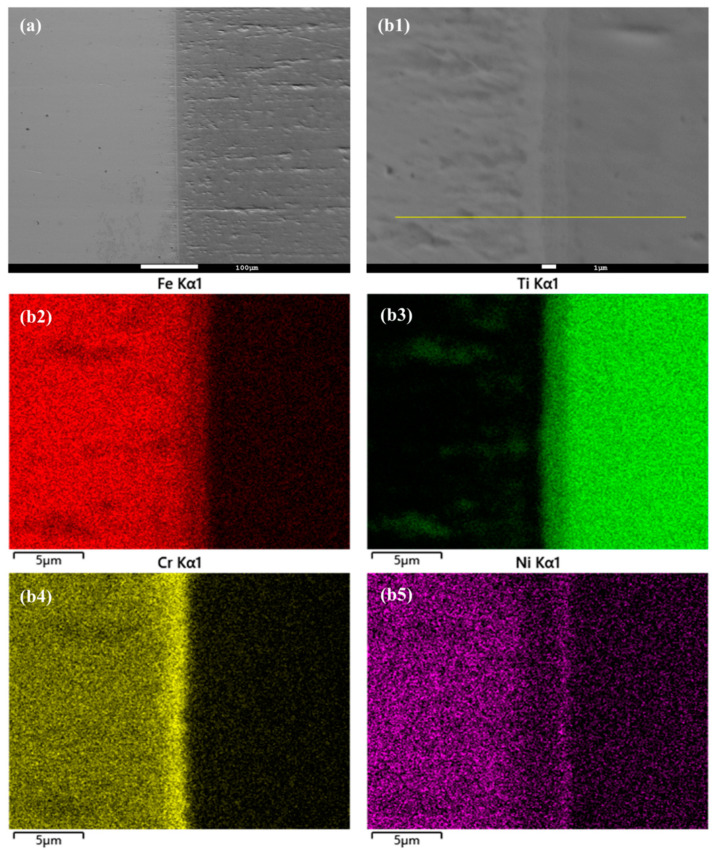
(**a**) Microstructure of Fe-Ti interface after HIP at 850 °C and 30 MPa for 40 min, and (**b1**–**b5**) higher magnification and EDS element mapping result.

**Figure 7:**
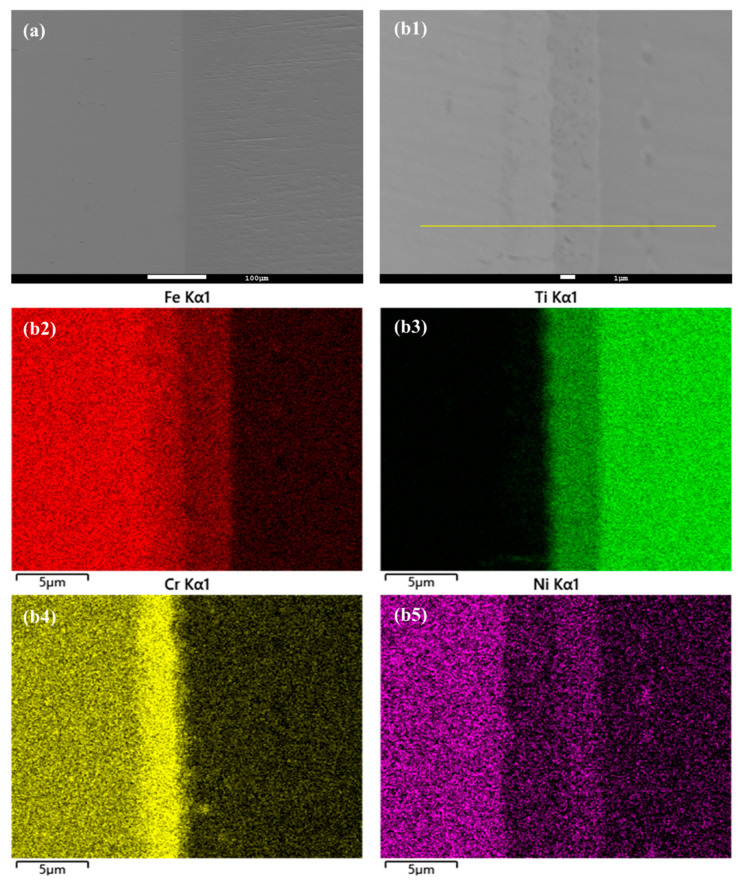
(**a**) Microstructure of Fe-Ti interface after HIP at 950 °C and 30 MPa for 40 min, and (**b1**–**b5**) higher magnification and EDS element mapping result.

In this study, the hot isostatic pressing (HIP) method was utilized for the diffusion welding process between Fe and Ti. The microstructure and EDS element mapping results of the Fe-Ti interface following the HIP process are presented in Figure 6 and Figure 7. Figure 6a and Figure 7a demonstrate a well-connected interface without any defects such as cracks between Fe and Ti. To further examine the microstructure and element distribution, Figure 6b displays a higher magnification image after heating at 850 °C and 30 MPa for 40 min. It is evident that Fe, Cr, and Ni elements are concentrated on the Fe side, while Ti is enriched on the other side. A smooth interface is observed, with two distinct sub-layers generated at the interface region. Based on the element distribution, these two sub-layers can be identified as a Fe-Cr-rich layer and a Ti-Ni-rich layer. Similarly, Figure 7b presents a similar result but with larger thicknesses for these two sub-layers. This finding confirms that increased temperature leads to higher diffusion rates in the diffusion couple.

**Figure 8:**
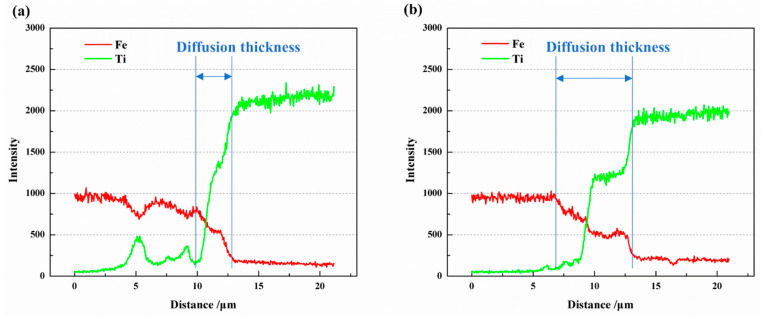
EDS line scan results (**a**) corresponding to the yellow line in Figure 6(b1), and (**b**) corresponding to the yellow line in Figure 7(b1).

The EDS line scan results of Fe and Ti atoms along the yellow lines in Figure 6(b1) and Figure 7(b1) are depicted in Figure 8. It is evident that a diffusion layer exists at the Fe/Ti interface, with the diffusion layer thickness being greater at 950 °C compared to 850 °C. At 850 °C, the diffusion layer thickness was 2.96 μm, while at 950 °C, it increased to 6.62 μm. Furthermore, the two sub-layers observed at the Fe-side and Ti-side can also be identified. As the temperature increased from 850 °C to 950 °C, the thickness of the sub-layer at the Fe-side increased from 0.87 μm to 2.47 μm, and the thickness at the Ti-side increased from 2.09 μm to 3.15 μm. It is worth highlighting that the sub-layer thickness at the Ti-side was greater than that at the Fe-side, indicating a faster diffusion rate from Fe to Ti compared to Ti to Fe. In a separate study by Li et al. [5], the formation of intermetallic compounds (IMCs) at the Fe/Ti interface was also observed. However, their work involved diffusion welding carbon steel and commercial pure titanium, revealing the presence of mostly TiC IMCs. Although they detected FeTi and Fe_2_Ti particles at the Fe/Ti interface, there was no plateau stage in element distribution at the interface, as observed in the present study.

### 3.3. Analysis on Diffusion Coefficient

**Figure 9:**
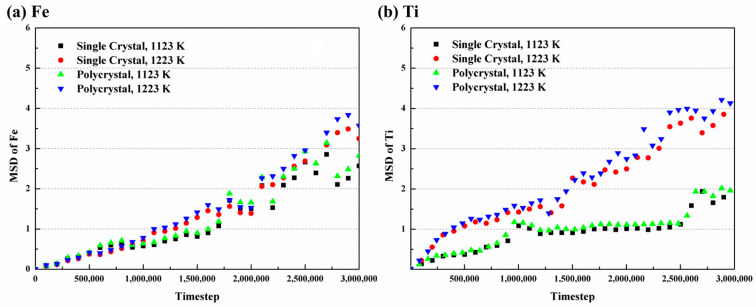
MSD curves with time of (**a**) Fe and (**b**) Ti atoms at different temperatures.

To analyze the diffusion behavior at Fe/Ti interface through MD simulations, the Einstein method was employed. Initially, the mean square displacement (MSD) of the atoms was obtained, allowing for the calculation of the MSD and diffusion coefficient D using the following formula:

(3) $$\underset{t\to \infty }{\lim}MSD=2dDt+c$$

(4) $$MSD=\frac{1}{N}\sum_{i=1}^{N}{\langle |r_{i}\left(t\right)-r_{i}\left(0\right)|}^{2}\rangle$$

(5) $$D=\frac{1}{6}\underset{t\to \infty }{\lim}\left(\frac{d}{dt}{\langle |r_{i}\left(t\right)-r_{i}\left(0\right)|}^{2}\rangle \right)$$

where d is the system dimension (3D in this study), t is the time, c is the constant, N is the atom number, and $r_{i}\left(t\right)$ is the displacement vector of i atom at t moment. Figure 9 illustrates the MSD curves at different conditions. The diffusion of Fe atoms shows relative stability throughout, while Ti atoms show as initially intense and gradually becoming more stable over time. Additionally, the curves of both atoms increase with rising temperature. Consequently, the diffusion coefficients in the simulations can be calculated.

For the diffusion welding experiments, the diffusion coefficient can also be obtained through the element distribution after diffusion. According to Fick’s law, the following equation can be obtained:

(6) $$\frac{\partial C}{\partial t}=D\frac{\partial^{2}C}{\partial x^{2}}$$

where C is the solute concentration, t is the time, and x is the distance along diffusion direction. For the diffusion couple, the error function of Equation (6) can be calculated by:

(7) $$C\left(x,t\right)=\frac{C_{2}+C_{1}}{2}-\frac{C_{2}-C_{1}}{2}erf\left(\frac{x}{2\sqrt{DT}}\right)$$

Therefore, the diffusion coefficient can be calculated based on the solute concentration distribution (Figure 8) and Equation (7).

**Figure 10:**
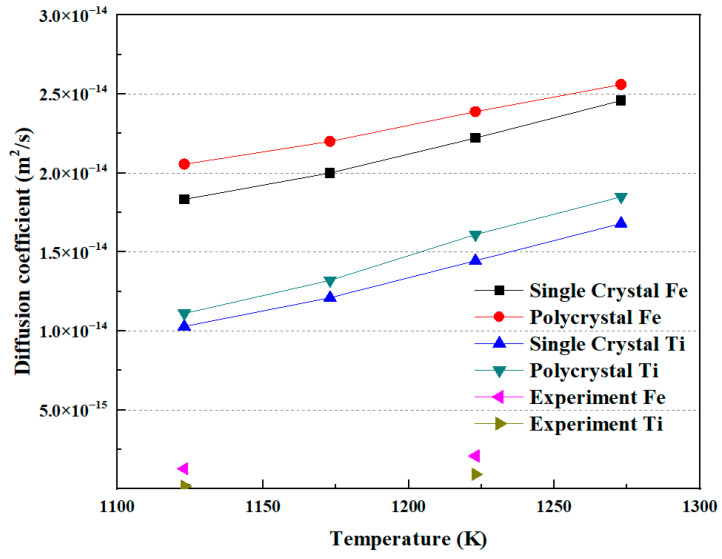
The correlation between diffusion coefficient and temperature.

As mentioned above, the diffusion coefficient results from both MD simulations and diffusion welding experiments have been obtained. Figure 10 illustrates the relationship between the diffusion coefficient and temperature. The diffusion coefficient exhibits a relationship with increasing diffusion temperature. Both Fe and Ti atoms show an increase in diffusion coefficient as the temperature rises, with Fe having a higher diffusion coefficient than Ti. Additionally, it can be observed that the diffusion coefficient in polycrystals is higher than that in a single crystal under the same conditions. In the experimental measurements, for Fe and Ti atoms, the diffusion coefficients of each at a diffusion temperature of 1123 K were 1.27 × 10^−15^ m^2^/s and 1.56 × 10^−16^ m^2^/s, respectively. At a diffusion temperature of 1223 K, the diffusion coefficients of each were 2.07 × 10^−15^ m^2^/s and 9.1 × 10^−16^ m^2^/s, respectively. These values are comparable to those obtained by Li et al. [5]. Xiang et al. [24] proposed that there is a temperature criterion for the diffusion between Fe and Ti. In this study, it can also be observed that the trend of the diffusion coefficient changing with temperature in the simulation results agrees well with the experimental results. However, the values of the diffusion coefficients in the experiments are less than those obtained in the MD simulation. The reason may be the existence of the gap, or noncontinuous interface, between Fe and Ti in the experiments.

## 4. Conclusions

In this study, molecular dynamic simulations and diffusion welding experiments were conducted to study the diffusion behavior at the Fe-Ti interface. The main conclusions are as follows:

(1) MD simulations of Fe-Ti joints at 1123 K and 1223 K show that the diffusion layer thickness is relatively larger at 1223 K compared to 1123 K. The diffusion layer becomes thicker with increasing diffusion time. The diffusion coefficients obtained from the MD simulations are at the level of 10^−14^ m^2^/s.
(2) Comparing the simulation results between single crystals and polycrystals, it is observed that the diffusion layer is thinner in a single crystal than in a polycrystal at the same temperature. This difference may be due to the presence of grain boundaries in polycrystals, which increase atomic disorder and facilitate diffusion.
(3) The RDF curves of Fe-Fe and Ti-Ti exhibit similar patterns at different times, showing a single main peak indicating the highest number density of atoms within that radius range and relatively strong bonding between the central atoms and their nearest neighbors. Increasing temperature leads to an increase in the peak value of Fe-Ti.
(4) The trend of the diffusion coefficient changing with temperature in the simulation results agrees well with the experimental findings. The disparity in the diffusion coefficient values between simulation and experiment may be attributed to the presence of gaps, or noncontinuous interfaces, between Fe and Ti in the experiments.

## Figures and Tables

**Table 1:** Chemical composition of 316 stainless steel (wt%).

Element	Cr	Ni	Mo	Mn	Si	C	Fe
Value	16.8	10.9	2.3	2.0	1.10	0.03	Balanced

## Data Availability

All data that support the findings of this study are available from the corresponding author upon reasonable request.
